# Exploring Mouse Protein Function via Multiple Approaches

**DOI:** 10.1371/journal.pone.0166580

**Published:** 2016-11-15

**Authors:** Guohua Huang, Chen Chu, Tao Huang, Xiangyin Kong, Yunhua Zhang, Ning Zhang, Yu-Dong Cai

**Affiliations:** 1 Department of Mathematics, Shaoyang University, Shaoyang, Hunan, 422000, China; 2 School of Life Sciences, Shanghai University, 99 Shangda Road, Shanghai, 200444, China; 3 Institute of Biochemistry and Cell Biology, Shanghai Institutes for Biological Sciences, Chinese Academy of Sciences, Shanghai, 200031, China; 4 Institute of Health Sciences, Shanghai Institutes for Biological Sciences, Chinese Academy of Sciences, Shanghai, 200031, China; 5 College of Life Science, Anhui Agricultural University, Hefei, Anhui, 230036, China; 6 Department of Biomedical Engineering, Tianjin Key Lab of Biomedical Engineering Measurement, Tianjin University, Tianjin, China; Cincinnati Children's Hospital Medical Center, UNITED STATES

## Abstract

Although the number of available protein sequences is growing exponentially, functional protein annotations lag far behind. Therefore, accurate identification of protein functions remains one of the major challenges in molecular biology. In this study, we presented a novel approach to predict mouse protein functions. The approach was a sequential combination of a similarity-based approach, an interaction-based approach and a pseudo amino acid composition-based approach. The method achieved an accuracy of about 0.8450 for the 1^st^-order predictions in the leave-one-out and ten-fold cross-validations. For the results yielded by the leave-one-out cross-validation, although the similarity-based approach alone achieved an accuracy of 0.8756, it was unable to predict the functions of proteins with no homologues. Comparatively, the pseudo amino acid composition-based approach alone reached an accuracy of 0.6786. Although the accuracy was lower than that of the previous approach, it could predict the functions of almost all proteins, even proteins with no homologues. Therefore, the combined method balanced the advantages and disadvantages of both approaches to achieve efficient performance. Furthermore, the results yielded by the ten-fold cross-validation indicate that the combined method is still effective and stable when there are no close homologs are available. However, the accuracy of the predicted functions can only be determined according to known protein functions based on current knowledge. Many protein functions remain unknown. By exploring the functions of proteins for which the 1^st^-order predicted functions are wrong but the 2^nd^-order predicted functions are correct, the 1^st^-order wrongly predicted functions were shown to be closely associated with the genes encoding the proteins. The so-called wrongly predicted functions could also potentially be correct upon future experimental verification. Therefore, the accuracy of the presented method may be much higher in reality.

## 1 Introduction

Recent advances in sequencing technology have identified a large number of proteins that perform a wide variety of functions in cellular activities. Knowledge of protein function is crucial to understanding the mechanisms behind cellular processes and preventing and treating disease. However, most of the proteins identified to date have unknown functions. Approximately 1% of the more than 13 million protein sequences available have been experimentally annotated with essential functions; the remaining proteins have been marked with putative, uncharacterized, hypothetical, unknown or inferred functions [1]. Although physical experimental approaches, including high-throughput screening, are capable of determining the biological functions of proteins, they are expensive and time-consuming. Additionally, these methods are aimed at certain functions, which produce one-sided descriptions of protein function [2].

Computational approaches can make up for the deficiencies of experiments. Following the success of the computational approach in sequence alignment and comparison, many computational techniques have been presented to determine protein functions during the last decade [3]. The most commonly applied approach is to transfer functional annotation from the most similar protein with known functional information. Both sequence and structural similarities are heavily utilized in this type of homology-based annotation transfer. To infer protein function, the servers OntoBlast [4] and GoFigure [5] use the sequence alignment tool BLAST [6]. Confunc [7], the protein function prediction (PFP) algorithm [8] and the extended similarity group method (ESG) [9] employ the sequence alignment tool PSI_BLAST [10]. The Blast2GO suite is the homology transfer-based functional annotation of the gene ontology vocabulary [11]. Similar to the sequence similarity-based method, the structure similarity-based approach generally uses structure alignments via programs such as DaliLite [12–14], STRUCTAL [15], MultiProt [16], Bioinfo3D [17], and 3DCoffee [18] to measure homology among proteins. PHUNCTIONER [19] utilizes structural alignment to identify crucial positions in a protein that might hold clues to specific functions. Pegg *et al*. [20] constructed a structure-function link database and used it to correct the errors in the annotation of enzymes. Some researchers have attempted to combine the sequence and structure similarity approaches to explore protein function. For example, the FRalanyzer [21] uses sequence-structure alignments to elucidate protein function.

Recently, a large body of protein-protein interaction networks has become available to explore the functional relationships between interacting proteins. There are many computational models for predicting protein-protein interactions [22–24]. The commonly accepted hypothesis (called guilt-by-association (GBA) [25]) is that proteins are more likely to share identical or similar functions with interacting proteins than with non-interacting proteins. Since Schwikowski *et al*. [26] pioneered the utility of interaction networks for annotating protein functions in yeast, numerous interaction-based methods have been proposed to infer the functions of proteins. Hishigaki *et al*. [27] presented an improved predictive method called the Chi-square method to elucidate protein function. Chi *et al*. [28] used an iterative strategy to transfer neighboring protein functions. Chua *et al*. [29] extended the neighborhood to indirect neighbors, called 2-neighbors. These types of local network predictions mainly transferred functional annotation from the directly interacting neighborhood. Additionally, some global optimization techniques have been adopted to elucidate protein function. For example, Deng *et al*. [30], Letovsky *et al*. [31] and Kourmpetis *et al*. [32] used the Bayesian Markov random field method to infer protein functions from protein-protein interaction data and functional annotation of the protein interaction partners. The protein-protein interaction network is viewed as a graph, where the nodes represent proteins and the edges represent the interactions between proteins. Some graph-based methods have been presented for function predictions. Nabieva *et al*. [33] modeled the functional annotation from the interaction network as a minimum multiway cut problem and introduced a network-flow algorithm that simulated the functional flow between proteins. The clustering-based and network alignment-based techniques have been employed to predict protein functions. Altaf-Ul-Amin *et al*. [34] and Arnau *et al*. [35] used different clustering techniques to classify protein functions, whereas Singh *et al*. [36] presented a global alignment of multiple protein interaction networks to infer protein functions. These approaches outperformed sequence similarity and local alignment of networks. Some researchers have presented a routine to predict protein function by combining multiple methods and data sources. For instance, Cozzetto *et al*. [2] integrated PSI-BLAST, text-mining, machine learning, and profile-profile comparisons to predict protein functions. As these authors noted, although considerable progress has been made, the functional annotation of integrative methods can be improved. Most of the above-mentioned networks are binary (*i*.*e*., 1 indicates interaction and 0 indicates no interaction). Additionally, the interaction between proteins can be strong or weak. The STRING database [37] is a protein interaction repository that characterizes each interaction into a weight value based on eight different lines of evidence. Hu *et al*. [38] used a weighted interaction to predict protein function and achieved a promising performance.

Great progress has been made in the computational protein function prediction field, where state-of-the-art prediction algorithms substantially outperform first-generation methods and contribute to subsequent experimental studies. However, there still remains considerable need for the improvement of the current tools [39]. To this end, we presented an integrated method to explore mouse protein functions by fusing sequence similarities, weighted interactions from the STRING database and the pseudo amino acid composition of proteins. Unannotated proteins were aligned against a database consisting of proteins with known functions. If the query protein was homologous to well-annotated proteins, the alignment scores were used to infer function. If there were no known homologous proteins, we extracted weighted interactions from the STRING database and used them to predict the query protein function. For proteins whose functions the previous two approaches could not predict, we used the pseudo amino acid composition (PseAAC)-based nearest-neighbor approach to elucidate their function.

## 2 Data and Methods

### 2.1 Data

A total of 14,732 mouse protein sequences with their functional annotations were downloaded from the Mouse Functional Genome Database (MfunGD, http://mips.gsf.de/genre/proj/mfungd/) [40], which is an important repository of protein sequences that provides high-quality protein function annotations with respect to cellular function exclusively for mice. To extensively examine the model for independency of homology, we used the sequence cluster program CD-HIT [41] to remove or reduce similarities between sequences. We obtained 12,478 proteins with a similarity threshold of 0.7. The mouse proteins in the MfunGD are annotated using the Functional Catalogue (FunCat) annotation scheme, which is widely used for the analysis of protein networks [42]. Compared with the GO categories, the FunCat category structure is simpler and more hierarchical.

As shown in **Table 1**, there are a total of 24 functional categories. The balance between the specificity of the categories, human usability and requirements for subsequent bioinformatic applications is a general consideration in the design of an annotation scheme [42]. In line with this notion, the 24-category-scheme for protein function classification is not performed at the most specific level, but it keeps our system descriptive and compact, which complies with the main goal of our study. The fact that the functions outnumber the proteins indicates that some proteins perform multiple functions. For details, see **S1 Table**.

**Table 1:** The number of mouse proteins in each category in our dataset.

Functional Number	Functional categories	Number of proteins
1	METABOLISM	2,401
2	ENERGY	522
3	CELL CYCLE AND DNA PROCESSING	971
4	TRANSCRIPTION	1,921
5	PROTEIN SYNTHESIS	399
6	PROTEIN FATE (folding modification destination)	2,187
7	PROTEIN WITH BINDING FUNCTION OR COFACTOR REQUIREMENT (structural or catalytic)	7,330
8	REGULATION OF METABOLISM AND PROTEIN FUNCTION	972
9	CELLULAR TRANSPORT TRANSPORT FACILITIES AND TRANSPORT ROUTES	2,078
10	CELLULAR COMMUNICATION/SIGNAL TRANSDUCTION MECHANISM	3,143
11	CELL RESCUE DEFENSE AND VIRULENCE	656
12	INTERACTION WITH THE ENVIRONMENT	1,212
13	SYSTEMIC INTERACTION WITH THE ENVIRONMENT	1,454
14	TRANSPOSABLE ELEMENTS VIRAL AND PLASMID PROTEINS	9
15	CELL FATE	1,180
16	DEVELOPMENT (Systemic)	939
17	BIOGENESIS OF CELLULAR COMPONENTS	769
18	CELL TYPE DIFFERENTIATION	317
19	TISSUE DIFFERENTIATION	313
20	ORGAN DIFFERENTIATION	491
21	SUBCELLULAR LOCALIZATION	8,467
22	CELL TYPE LOCALIZATION	232
23	TISSUE LOCALIZATION	261
24	ORGAN LOCALIZATION	542
Total	—	38,766

Protein-protein interaction pairs in mice were retrieved from STRING (Version 9.1, http://string-db.org/) [37], which is a protein-protein interaction database that collects known or predicted, direct (physical) or indirect (functional) associations. STRING quantifies each pair of protein interactions into a combined score. Currently, STRING contains 5,214,234 proteins from 1,133 organisms.

Because the manner in which the entries in the MfunGD are numbered differs from the method in STRING, comparison requires the mapping of associations between them. The mapping was performed using the BioMart database [43]. A total of 10,539 of the 12,478 proteins in MfunGD were mapped to the proteins in STRING.

### 2.2 Methods

The aim of this study is to predict the function of a given protein *P* based on *n* known-function proteins *P*_1_, *P*_2_, …, *P*_*n*_, assuming that the function categories are *f*_1_, *f*_2_, …, *f*_24_. One protein may belong to several function categories (*e*.*g*., the protein mc10000007 belongs to categories *f*_8_ ‘REGULATION OF METABOLISM AND PROTEIN FUNCTION’ and *f*_10_ ‘CELLULAR COMMUNICATION/SIGNAL TRANSDUCTION MECHANISM’). Thus, we used a 24-dimensional vector *F*_*i*_ *=* (*d*_1,*i*_, *d*_2,*i*_, …, *d*_24,*i*_) to indicate the function categories of a protein *P*_*i*_, where *d*_*j*,*i*_ is

$$d_{j,i}=\{\begin{array}{cc} 1 & P_{i}\;\text{has function}\;f_{j}\\ 0 & P_{i}\;\text{does not have function}\;f_{j} \end{array}$$ (1)

Three methods were used in this study to achieve this goal.

### 2.2.1 Sequence similarity-based approach

Proteins with similar sequences likely share the same or similar functions. Therefore, it is possible to predict protein functions based on sequence similarities. Herein, we used the PSI-BLAST program (E-value 0.001, iteration 3) to align the given unknown-function protein (*P*) against the known-function proteins (*P*_1_, *P*_2_, …, *P*_*n*_) in our dataset. The alignment score between *P* and *P*_*i*_ represents their similarity. This score is denoted as *s*_*i*_. The predicted protein function scores of protein *P* are given by a 24-dimensional vector *W* and are calculated by

$$W=\left[\begin{array}{c} w_{1}\\ w_{2}\\ \vdots \\ w_{24} \end{array}\right]=\left[\begin{array}{cccc} d_{1,1} & d_{1,2} & \ldots & d_{1,n}\\ d_{2,1} & d_{2,2} & \ldots & d_{2,n}\\ \vdots & \vdots & \vdots & \vdots \\ d_{24,1} & d_{24,2} & \ldots & d_{24,n} \end{array}\right]\left[\begin{array}{c} s_{1}\\ s_{2}\\ \vdots \\ s_{n} \end{array}\right]$$ (2)

where *w*_*j*_ denotes the score of a protein having function *f*_*j*_. Elements in vector *W* are sorted from highest to lowest to obtain the predicted functions of protein *P*. A function receiving a high score is more likely to be an actual function of a given protein according to GBA [44] because there are several known proteins similar to the given protein that have this function. Thus, a function sequence can be constructed according to *W*. We provide an example to elaborate this point. If we obtain *w*_23_ ≥ *w*_2_ ≥…≥ *w*_5_, protein *P* is most likely to have function *f*_23_, followed by function *f*_2_ and so forth. The least likely function is *f*_5_. For convenience, we call function *f*_23_ the 1^st^-order prediction, function *f*_2_ the 2^nd^-order prediction and function *f*_5_ the 24^th^-order prediction. This scheme to define the predicted results for multi-label classification problems has been used in previous studies [38, 45, 46].

#### 2.2.2 Weighted interaction-based approach

Proteins in a cell interact with each other to perform particular functions. Following the GBA rule [25], interacting proteins may possess similar functions. We used the combined scores in the STRING database as weighted values between proteins. These values represent a fusion of eight types of evidence, including co-expression, gene fusion and experimental evidence. We assume that the combined score between *P* and *P*_*i*_ (*i* = 1,2,⋯,*n*) is *t*_*i*_. The predictive functional value is given by Y, a 24-dimensional vector computed by

$$Y=\left[\begin{array}{c} y_{1}\\ y_{2}\\ \vdots \\ y_{24} \end{array}\right]=\left[\begin{array}{cccc} d_{1,1} & d_{1,2} & \ldots & d_{1,n}\\ d_{2,1} & d_{2,2} & \ldots & d_{2,n}\\ \vdots & \vdots & \vdots & \vdots \\ d_{24,1} & d_{24,2} & \ldots & d_{24,n} \end{array}\right]\left[\begin{array}{c} t_{1}\\ t_{2}\\ \vdots \\ t_{n} \end{array}\right]$$ (3)

where *y*_*j*_ denotes the score of a given protein with function *f*_*j*_. Similar to the sequence similarity-based approach, each element *y*_*j*_ in vector *Y* is sorted from highest to lowest to obtain the function sequence of protein *P*. For example, if we obtain *y*_12_ ≥ *y*_21_ ≥…≥ *y*_2_, protein *P* is most likely to have function *f*_12_, followed by *f*_21_, and the least likely function is *f*_2_. In this study, we call function *f*_12_ the 1^st^-order prediction, function *f*_21_ the 2^nd^-order prediction, and function *f*_2_ the 24^th^-order prediction.

#### 2.2.3 PseAAC-based approach

Protein sequences can be characterized by pseudo amino acid composition, which was proposed by Chou to predict protein subcellular localization [47] and has become popular in the prediction of post-translational modification sites [48, 49] and membrane protein types [50–52]. PseAAC maps a protein sequence into a numerical vector. If a protein sequence is *X*_1_*X*_2_⋯*X*_*N*_, where *X*_*i*_ is an amino acid residue, then *L*(*X*) is the property value of amino acid *X* in the physicochemical and biochemical respects. The normalized property value is computed by

$$F(X)=\frac{L(X)-\frac{1}{20}\sum_{Y\in \Phi }^{}L(Y)}{\sqrt{\sum_{X\in \Phi }(L(X)-\frac{1}{20}\sum_{Y\in \Phi }L(Y){)}^{2}/20}}$$ (4)

where Φ is the set of 20 types of amino acids. The correlation factor between residues in the protein sequence is computed by

$$C_{i}=\frac{1}{N-i}\sum_{k=1}^{N-i}((F(X_{k})-F(X_{k+i}){)}^{2},\;i=1,2,\cdots ,\lambda ,\;\lambda <N$$ (5)

The correlation factors reflect information about the position and category of amino acids in the protein sequence. The PseAAC of a protein sequence is computed by

$$v_{i}=\{\begin{array}{cc} \frac{f_{i}}{\sum_{j=1}^{20}f_{j}+\varpi \sum_{k=1}^{\lambda }C_{k}} & 1\leq i\leq 20\\ \frac{\varpi C_{i-20}}{\sum_{j=1}^{20}f_{j}+\varpi \sum_{k=1}^{\lambda }C_{k}} & 21\leq i\leq \lambda +20 \end{array}$$ (6)

where ϖ is the sequence order effects, and *f*_*i*_ is the occurrence frequency of amino acids. In this article, we set λ and ϖ to 50 and 0.15, respectively. Five physicochemical and biochemical properties of amino acids, i.e., codon diversity, electrostatic charge, molecular volume, polarity and secondary structure, are used to compute the PseAAC of protein sequences. These properties are retrieved from references [53–55], as listed in **Table 2**. For each category of property, we used the last 50 digits in the formula (6). In addition to the frequencies of 20 amino acids, we used a 270 (20+5*50)-dimensional vector to represent a protein sequence. The cosine distance between the query protein *P* and the known-function proteins *P*_*i*_ is given by

$$\Delta (P_{i},P)=\frac{V_{p}•V_{P_{i}}}{\left\|V_{p}\right\|\left\|V_{P_{i}}\right\|}$$ (7)

where the operators • and ‖ ‖ indicate the inner product and module of vectors, respectively, and *V*_*P*_ and $V_{P_{i}}$ are the 270-dimensional PseAACs of proteins *P* and *P*_*i*_, respectively. The predicted function value of the query protein was computed by

$$R=\left[\begin{array}{c} r_{1}\\ r_{2}\\ \vdots \\ r_{24} \end{array}\right]=\left[\begin{array}{cccc} d_{1,1} & d_{1,2} & \ldots & d_{1,n}\\ d_{2,1} & d_{2,2} & \ldots & d_{2,n}\\ \vdots & \vdots & \vdots & \vdots \\ d_{24,1} & d_{24,2} & \ldots & d_{24,n} \end{array}\right]\left[\begin{array}{c} \Delta (P_{1},P)\\ \Delta (P_{2},P)\\ \vdots \\ \Delta (P_{n},P) \end{array}\right]$$ (8)

Similar to the two above approaches, the elements in the vector *R* are sorted from high to low, such as *r*_3_ > *r*_12_ > ⋯ > *r*_1_, where protein *P* is most likely to have function *f*_3_, second most likely to have function *f*_12_, and least likely to have function *f*_1_.

**Table 2:** The physicochemical and biochemical properties of the 20 amino acids.

Amino acid	Polarity	Second structure	Molecular volume	Codon diversity	Electrostatic charge
A	-0.591	-1.302	-0.733	1.57	-0.146
C	-1.343	0.465	-0.862	-1.02	-0.255
D	1.05	0.302	-3.656	-0.259	-3.242
E	1.357	-1.453	1.477	0.113	-0.837
F	-1.006	-0.59	1.891	-0.397	0.412
G	-0.384	1.652	1.33	1.045	2.064
H	0.336	-0.417	-1.673	-1.474	-0.078
I	-1.239	-0.547	2.131	0.393	0.816
K	1.831	-0.561	0.533	-0.277	1.648
L	-1.019	-0.987	-1.505	1.266	-0.912
M	-0.663	-1.524	2.219	-1.005	1.212
N	0.945	0.828	1.299	-0.169	0.933
P	0.189	2.081	-1.628	0.421	-1.392
Q	0.931	-0.179	-3.005	-0.503	-1.853
R	1.538	-0.055	1.502	0.44	2.897
S	-0.228	1.399	-4.76	0.67	-2.647
T	-0.032	0.326	2.213	0.908	1.313
V	-1.337	-0.279	-0.544	1.242	-1.262
W	-0.595	0.009	0.672	-2.128	-0.184
Y	0.26	0.83	3.097	-0.838	1.512

### 2.3 Cross-Validation and Assessment

We used two cross-validation methods: leave-one-out cross-validation and ten-fold cross-validation to examine the performance of the presented methods. In the ten-fold cross-validation method, the original dataset are randomly and equally divided into ten parts. Samples in each part are singled out as testing samples, while samples in other nine parts are used as training samples. For the leave-one-out cross-validation approach, each sample in the original dataset is taken as a testing sample in turn and the remaining samples are used as training samples. To assess the experimental results, the prediction accuracy for the *j*^th^-order prediction is given by

$$ACC_{j}=\frac{1}{n}\sum_{i=1}^{n}U_{j,i},\;j=1,2,\cdots ,24$$ (9)

where *U*_*j*,*i*_ = 1 if the function category of the *j*^th^-order prediction is actually the function category of protein *P* according to current knowledge. Otherwise, *U*_*j*,*i*_ = 0.

## 3 Results and Discussion

### 3.1 Performance of the Simple Approach

The performance of the three approaches for a dataset consisting of 12,478 proteins evaluated by the leave-one-out method is listed in **Table 3**. The similarity-based approach yielded the best accuracy of 0.8756 in the 1^st^-order prediction but could not predict functions of 2,226 proteins because they have no homologues with annotated proteins in the dataset. The interaction-based approach produced a lower prediction accuracy of 0.7535 than the similarity-based approach and could not predict the functions of 1,939 proteins that have no interactions with annotated proteins. The PseAAC-based nearest-neighbor approach performed worst in terms of the prediction accuracy, but it was able to predict the functions of all the test proteins. The results indicated that each approach has its strengths and limitations. **Table 3**also shows that the 1^st^-order prediction performed best, followed by the 2^nd^-order prediction and the 3^rd^-order prediction, indicating the predicted function sequence for each test protein is quite reasonable.

**Table 3:** Prediction accuracies of three methods and the combined method in the first three order predictions.

Method	Number of proteins of testing dataset	1^st^-order	2^nd^-order	3^rd^-order
Similarity-based	10,252	0.8756	0.7132	0.5158
Interaction-based	10,539	0.7535	0.6296	0.5299
PseAAC-based	12,478	0.6786	0.5874	0.2519
Combined	12,478	0.8464	0.6814	0.4996

**Fig 1:**
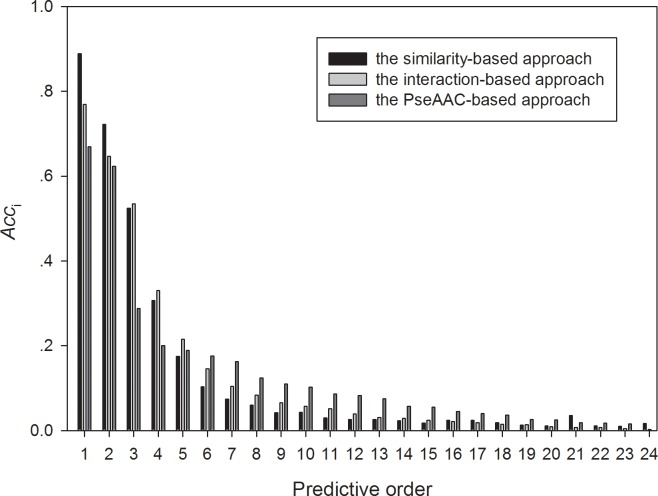
The prediction accuracies of 24 order predictions for these three methods on the common dataset.

The three approaches were compared on different testing datasets in the above paragraph. For a fair comparison, we generated a common dataset where each protein could be tested by the leave-one-out method. The common dataset consisted of 8,481 proteins. The accuracies of the three approaches versus order are plotted in **Fig 1**. The similarity-based approach performed best, followed by the interaction-based approach and the PseAAC-based nearest-neighbor approach. The similarity-based approach was much more accurate (by 0.11) than the interaction-based approach in the 1^st^-order prediction and more accurate (by 0.07) in the 2^nd^-order prediction, while the latter was much more accurate (by 0.09) than the PseAAC-based approach in the 1^st^-order prediction and more accurate (by 0.02) in the 2^nd^-order prediction. The results confirmed the advantage of the similarity-based approach over the other two approaches in terms of the prediction accuracy. As mentioned previously, the similarity-based approach cannot address non-homologous proteins, and the PseAAC-based approach can predict the functions off all proteins despite the lower prediction accuracy. Therefore, it is wise to jointly utilize the three methods to predict the protein functions.

### 3.2 Prediction by the Combined Approach

We combined the three approaches to predict the functions of proteins to make use of their respective advantages and disadvantages. For a given protein, we first employed the similarity-based approach. If the protein had no homologues, we applied the interaction-based approach. If the protein could not be predicted by the interaction-based approach, we used the PseAAC-based nearest-neighbor approach. The performance of the combined approach based on leave-one-out validation on the 12,478 proteins is shown in the fifth row of **Table 3**. The accuracy of the combined method was much higher than the interaction-based and PseAAC-based approaches and slightly lower than the similarity-based approach. However, the combined approach could predict all proteins, whereas the similarity-based approach could not. Therefore, the combined method has wide application at the cost of reduced prediction accuracy. For proteins with no homologues or interactions with annotated proteins, the best alternative is to use the combined approach. The contributions of the three approaches to the final predictive performance are shown in **Table 4**. The similarity-based approach contributed most, predicting more than 80% of all proteins and yielding an *Acc*_1_ of 0.8756, followed by the interaction-based approach and the PseAAC-based approach.

**Table 4:** Contributions of the three approaches to the predicted results.

Method	Number of proteins	Proportion	*Acc*_1_
Similarity-based approach	10,252	82.16%	0.8756
Interaction-based approach	1,876	15.03%	0.7154
PseAAC-based approach	350	2.81%	0.6943

To fully indicate the effectiveness of the combined method, we also used ten-fold cross-validation to examine this method. Because the predicted results yielded by this cross-validation method may influenced by the division of the dataset, the combined method was executed five times with different divisions. The prediction accuracies for the 1^st^-order, 2^nd^-order and 3^rd^-order predictions in each time are listed in **Table 5**. Compared to the prediction accuracies yielded by the leave-one-out cross-validation that are listed in **Table 3**, the performances of these two cross-validation methods are almost at the same level, which indicates that the combined method is still quite effective when there are no close homologs are available. Furthermore, it can be observed from **Table 5**that the standard deviations for the 1^st^-order, 2^nd^-order and 3^rd^-order predictions are quite low, indicating the stability of the combined method.

**Table 5:** Performances of the combined method evaluated by ten-fold cross validation.

Order	1	2	3	4	5	Mean ± std ^a^
1st	0.8429	0.8416	0.8420	0.8440	0.8419	0.8425 ±0.0010
2nd	0.6768	0.6792	0.6781	0.6787	0.6802	0.6786 ±0.0013
3rd	0.5023	0.4972	0.5007	0.4977	0.4998	0.4995 ±0.0021

a: std is the abbreviation of standard deviation.

### 3.3 Possible Protein Functions

In this study, the assessment of the predicted results was based on currently annotated proteins. Therefore, "right" and "wrong" predictions were relatively defined. For example, if the studied protein had function *F*_*A*_ and the predicted function was *F*_*B*_, the prediction was not correct. It is conceivable that with the development of our knowledge, the protein could be found to possess *F*_*B*_; thus, the prediction could be correct in the future. The currently annotated functions of the proteins are a subset of their actual functions. In this respect, some "wrong" predictions by our method in the current dataset may be correct. Next, we explore these wrong predictions.

It is worth performing further analysis on the wrongly predicted proteins. Because the 1^st^-order prediction is the most important, we investigated proteins with "wrong" 1^st^-order prediction but with "right" 2^nd^-order prediction. Because these proteins might possess the predicted 1^st^-order functions, we called them "false-wrong" 1^st^-order predicted proteins. As mentioned above, the combined method was evaluated by both the leave-one-out and ten-fold cross-validations. Because the predicted results yielded by the ten-fold cross-validations are not unique, we selected the predicted results yielded by the leave-one-out cross-validation to further analyze wrongly predicted proteins. In the leave-one-out test on the 12,478 proteins, we identified 966 such proteins: 658 proteins from the similarity-based approach, 258 proteins from the interaction-based approach and 50 proteins from the PseAAC-based approach. All these proteins are listed in **S2 Table**.

The goal of this process was to further validate our method. If we found evidence indicating that any of these proteins possessed the "wrong-predicted" functions, the actual prediction accuracy of our method would be much higher than presented above. This would allow the method to be applied to new protein function discoveries, but further experimental validations may be required for these proteins.

### 3.4 Possible Function Analysis of Significant "False-Wrong" 1st-Order Predicted Proteins

We explored the functions of proteins whose predicted 1^st^-order functions were wrong and whose predicted 2^nd^-order functions were correct. There were 966 such proteins. Forty protein genes were closely related to "false-wrong" 1st-order predicted functions, of which sixteen were predicted by the similarity-based approach, twenty-two were predicted by the interaction-based approach, and two were predicted by the PseAAC-based approach, as listed in **Table 6**, **Table 7**and **Table 8**, respectively.

**Table 6:** The sixteen significant proteins with "wrong" 1^st^-order predictions but "right" 2^nd^-order predictions based on the sequence similarity-based approach.

Protein ID	Name	"wrong" predicted function in 1st-order prediction
mc11000118	MYO1G	SUBCELLULAR LOCALIZATION
mc9001073	NEO1	SUBCELLULAR LOCALIZATION
mc5002204	SDK1	SUBCELLULAR LOCALIZATION
mc17000153	PLG	PROTEIN FATE (folding, modification, destination)
mc2000415	GM711	PROTEIN FATE (folding, modification, destination)
mc15000840	MAPK15	PROTEIN FATE (folding, modification, destination)
mc7000273	PRKD2	PROTEIN FATE (folding, modification, destination)
mc11002342	STRADA	PROTEIN FATE (folding, modification, destination)
mc7001424	NTRK3	PROTEIN FATE (folding, modification, destination)
mc14000439	BMPR1A	PROTEIN FATE (folding, modification, destination)
mc11001586	KSR1	PROTEIN FATE (folding, modification, destination)
mc6000496	EPHB6	PROTEIN FATE (folding, modification, destination)
mc7000874	KLK9	PROTEIN FATE (folding, modification, destination)
mc15001663	KRT2	PROTEIN WITH BINDING FUNCTION OR COFACTOR REQUIREMENT (structural or catalytic)
mc17001082	PTK7	PROTEIN WITH BINDING FUNCTION OR COFACTOR REQUIREMENT (structural or catalytic)
mc1000962	SPEG	PROTEIN WITH BINDING FUNCTION OR COFACTOR REQUIREMENT (structural or catalytic)

**Table 7:** The twenty-two significant proteins with "wrong" 1^st^-order predictions but "right" 2^nd^-order predictions based on the weighted interaction-based approach.

Protein ID	Name	"wrong" predicted function in 1st-order prediction
mc2003319	ADRM1	SUBCELLULAR LOCALIZATION
mc6000275	ATP6V1F	SUBCELLULAR LOCALIZATION
mc4002507	AURKAIP1	SUBCELLULAR LOCALIZATION
mc17001119	BYSL	SUBCELLULAR LOCALIZATION
mc13001367	DHFR	SUBCELLULAR LOCALIZATION
mc1001293	DTYMK	SUBCELLULAR LOCALIZATION
mc4000473	GNE	SUBCELLULAR LOCALIZATION
mc5001787	HPD	SUBCELLULAR LOCALIZATION
mc3000151	HPS3	SUBCELLULAR LOCALIZATION
mc4001314	MAGOH	SUBCELLULAR LOCALIZATION
mc9000131	MED17	SUBCELLULAR LOCALIZATION
mc4001915	NUDC	SUBCELLULAR LOCALIZATION
mc11000229	PNOL	SUBCELLULAR LOCALIZATION
mcx000234	RGN	SUBCELLULAR LOCALIZATION
mc9000734	RPS25	SUBCELLULAR LOCALIZATION
mc8000054	SHCBP1	SUBCELLULAR LOCALIZATION
mc6000048	SHFM1	SUBCELLULAR LOCALIZATION
mc2002263	NCAPH	PROTEIN WITH BINDING FUNCTION OR COFACTOR REQUIREMENT (structural or catalytic)
mc2000861	RIF1	PROTEIN WITH BINDING FUNCTION OR COFACTOR REQUIREMENT (structural or catalytic)
mc19000070	CDCA5	PROTEIN WITH BINDING FUNCTION OR COFACTOR REQUIREMENT (structural or catalytic)
mc7001471	PRC1	PROTEIN WITH BINDING FUNCTION OR COFACTOR REQUIREMENT (structural or catalytic)
mc15001589	NPFF	CELLULAR COMMUNICATION/SIGNAL TRANSDUCTION MECHANISM

**Table 8:** The two significant proteins with "wrong" 1^st^-order predictions but "right" 2^nd^-order predictions based on the PseAAC-based approach.

Protein ID	Name	"wrong" predicted function in 1st-order prediction
mc4000691	AKAP2	SUBCELLULAR LOCALIZATION
mc1001669	KISS1	SUBCELLULAR LOCALIZATION

As shown in **Table 6**, sixteen significant proteins were predicted by the similarity-based approach. The proteins MYO1G, NEO1 and SDK1 were predicted to have the 1^st^-order function ‘subcellular localization’, suggesting that these gene products have specific cellular localizations. MYO1G has been reported as a hematopoietic-specific myosin that localizes to the plasma membrane [56]. Moreover, neogenin 1 (NEO1) and sidekick cell adhesion molecule 1 (SDK1) are likely to localize on the plasma membrane based on their biological functions. The proteins PLG, GM711, MAPK15, PRKD2, STRADA, NTRK3, BMPR1A, KSR1, EPHB6 and KLK9 were predicted to have the 1^st^-order function ‘protein fate (folding/modification/destination)’. MAPKs, BMP, KSR1, PRKD2, STRADA, NTRK3 and EPHB6 are responsible for protein phosphorylation and signal transduction [57–63]. KLK9 belongs to the family of kallikrein-related peptidases (KLKs), which possess trypsin-like proteolytic activity [64, 65]. Plasminogen (PLG) is a precursor of the key enzyme of the fibrinolytic system plasmin, which serves as a physiological backup enzyme for ADAMTS13 (a disintegrin and metalloproteinase with a thrombospondin type I motif, member 13) in the degradation of pathological platelet-VWF (Von Willebrand factor) complexes [66]. KRT2, PTK7 and SPEG were predicted to have the 1^st^-order function ‘protein with binding function or cofactor requirement’. Protein tyrosine kinase 7 (PTK7) was reported to interact with the Wnt family proteins [67] and play a pivotal role in planar cell polarity [68]. The intermediate filament keratin proteins, including Keratin 2 (KRT2), bind and interact with signaling molecules, such as CFTR [69], trichoplein [70] and Albatross complexes [71]. SPEG complex locus (SPEG) is a myotubularin (MTM1)-binding protein, and its deficiency has been proven to cause centronuclear myopathy with dilated cardiomyopathy [72].

As shown in **Table 7**, twenty-two significant proteins were predicted by the interaction-based approach. ADRM1, ATP6V1F, AURKAIP1, BYSL, DHFR, DTYMK, GNE, HPD, HPS3, MAGOH, MED17, NUDC, PNO1, RGN, RPS25, SHCBP1 and SHFM1 were predicted to have the 1^st^-order function ‘subcellular localization’. ATPase, H^+^ transporting, lysosomal 14 kDa, V1 subunit F (ATP6V1F) and adhesion regulating molecule 1 (ADRM1) are likely to localize on the plasma membrane based on their biological functions. Several gene products are specifically localized in the nucleus, including AURKAIP1, DHFR, MAGOH, MED17, NUDC, PNO1 and RGN. Among them, NUDC is a nuclear movement protein that interacts with dynein [73]. Mediator complex subunit 17 (MED17) is localized in the nucleus and is involved in transcription regulation [74, 75]. The Bystin-like (BYSL) protein was reported to colocalize with trophinin, tastin and cytokeratins in the cytoplasm, forming a complex in trophectoderm cells that is essential for embryo implantation and ribosomal biogenesis [76]. The ribosomal protein S25 (RPS25) is also located in the cytoplasm and is responsible for protein synthesis [77]. The protein 4-hydroxyphenylpyruvate dioxygenase (HPD) is enriched in the liver cell cytoplasm and encodes an enzyme involved in the catabolic pathway of tyrosine, which catalyzes the conversion of 4-hydroxyphenylpyruvate to homogentisate [78]. SHFM1 (split hand/foot malformation (ectrodactyly) type 1, also known as DSS1) localizes to proteasomes [79]. Additionally, we predicted the specific subcellular localization of Hermansky-Pudlak syndrome 3 (HPS3), which encodes a novel protein with largely unknown function [80], together with aurora kinase A interacting protein 1 (AURKAIP1), deoxythymidylate kinase (DTYMK), glucosamine (UDP-N-acetyl)-2-epimerase/N-acetylmannosamine kinase (GNE), partner of NOB1 homologue (PNO1), dihydrofolate reductase (DHFR), and SHC SH2-domain binding protein 1 (SHCBP1). Our data provide clues for the future study of these genes. NCAPH, RIF1, CDCA5 and PRC1 were predicted to have the 1^st^-order function ‘protein with binding function or cofactor requirement’. NCAPH (also known as CAP-H) binds to the chromosome and regulates the cell cycle [81]. CDCA5 (also known as SORORIN) binds to sister chromatids and regulates their separation [82]. Protein regulator of cytokinesis 1 (PRC1) was shown to bind to several motor proteins, including KIF4, MKLP1 and CENP-E, and play pivotal roles in the formation of microtubule architecture [83]. Replication timing regulatory factor (RIF1) is responsible for regulating the replication-timing program in mammalian cells [84]. It was shown to bind to aberrant telomeres and to align along the anaphase midzone microtubules [85]. NPFF was predicted to have the 1^st^-order function ‘cellular communication’. NPFF (neuropeptide FF) is an FMRFamide-like peptide with antiopiate properties that is involved in cellular communication as a part of the neurotransmitter system [86, 87].

As shown in **Table 8**, two significant proteins were predicted by the PseAAC-based approach. A-kinase anchor protein 2 (AKAP2) has the known function of ‘protein fate (folding, modification, destination)’ as it regulates cyclic AMP-dependent protein kinase (PKA) signaling in both a spatial and temporal manner. The specific subcellular localization of AKAP2 is closely related to its function [88]. AKAP2 has both cytosolic and endosomal localizations, and a fraction of endosomal AKAP2 is involved in regulating the expression of several downstream proteins, such as Rab4 and Rab11, and endosomal functions [89]. As another example, kisspeptins (KISS1) have known functions related to ‘protein with binding function or cofactor requirement.’ The versatile and complex pathways of KISS1 and their receptors play essential roles in the development of the brain and the reproductive system [90] and induce apoptosis in various cancers [91, 92]. Previous publications have shed light on both the cytosolic and nuclear localization of KISS1 receptors, which were linked to distinct functions, such as cytosolic calcium elevation and potential nuclear transactivation activity [93, 94]. These lines of evidence support our prediction of the important ‘subcellular localization’ function of these proteins.

## 4. Conclusion

The accurate identification of protein functions remains challenging in the post-genomic era. In this article, we employed protein sequence homology, weighted interactions and pseudo amino acid composition to explore protein functions. The experimental results indicate that homologous proteins are more likely to share functions than interacting proteins, which in turn share more functions than proteins with similar physicochemical and biochemical properties. Weighted interactions can be used to annotate the functions of proteins with no known homologues. The PseAAC-based approach was used for the functional annotation of proteins. These three approaches are complementary and represent an optimal combination for predicting protein functions. Further analyses of wrongly predicted functions will validate the effectiveness of the proposed method.

## Supporting Information

S1 Table The dataset used in this study. The first column of the file is the protein entry ID in MfunGD. The other columns are the functional categories to which the protein belongs.
(CSV)

S2 Table The "false-wrong" 1^st^-order predicted proteins. Proteins with "wrong" 1^st^-order function predictions but "right" 2^nd^-order function predictions in our dataset were called "false-wrong" 1^st^-order predicted proteins. There were 658 such proteins based on the similarity-based approach, 258 based on the interaction-based approach and 50 based on the PseAAC-based approach. These proteins are listed on three separate sheets. The proteins may possess the function indicated by the 1st-order prediction and are worthwhile subjects for future analysis.
(XLSX)
